# Effects of yeast culture (*Saccharomyces cerevisiae*) on growth performance, serum biochemistry, rumen fermentation and microbiota of intake-restricted multiparous Suffolk sheep

**DOI:** 10.3389/fmicb.2025.1601805

**Published:** 2025-08-11

**Authors:** Chuying Wang, Yujie Niu, Peng Zhang, Qicheng Lu, Jingquan Yang, Ning Chen, Wenju Zhang

**Affiliations:** ^1^Animal Nutrition and Feed Science, College of Animal Science and Technology, Shihezi University, Shihezi, China; ^2^Xinjiang Academy of Agricultural and Reclamation Science, Shihezi, China

**Keywords:** intake restriction, yeast culture, rumen fermentation, rumen microbiota, growth performance

## Abstract

**Introduction:**

This study evaluated the effects of yeast culture (YC) supplementation on growth performance, nutrient digestibility, serum parameters, rumen fermentation, and bacterial communities in intake-restricted multiparous Suffolk sheep, aiming to provide a theoretical basis for enhancing productivity.

**Methods:**

Thirty multiparous Suffolk sheep (Suffolk♂ × Hu♀), with a mean body weight of 22 ± 0.5 kg, were arbitrarily assigned to three experimental groups: ad libitum feeding (AL), intake restriction (20% reduction, IR), and intake restriction with 30 g/d YC supplementation (20% reduction, IRY) groups (*n* = 10), and each sheep was housed separately.

**Results:**

The findings indicated that, compared to the IR group, the IRY group exhibited significantly increased average daily gain (ADG), net weight gain (NWG), digestibility of neutral detergent fiber (NDF) and acid detergent fiber (ADF), pH, total protein (TP), glucose (GLU), propionate, and immunoglobulin A (IgA) (*p* < 0.05), while feed-to-gain ratio (F/G) and acetate: propionate ratio (A: P) were significantly decreased (*p* < 0.05). Furthermore, differential feeding methods have significantly changed the composition of ruminal microbiota. The Shannon and Simpson indices were significantly higher in the IR and IRY groups compared with those in the AL group (*p* < 0.05), and the Chao1 index in the IRY group was significantly higher than that in the AL group (*p* < 0.05). The relative abundance of *Prevotella* in the IR group was significantly lower than that in the AL group (*p* < 0.05). The relative abundance of *Ruminococcus* in the IR and IRY groups was significantly lower than that in the AL group (*p* < 0.05). In contrast, the relative abundance of *Bifidobacterium* and *Butyrivibrio* was significantly higher in the IRY group compared with that in the AL and IR groups (*p* < 0.05).

**Discussion:**

These results indicate that YC supplementation under intake restriction improves growth performance by enhancing apparent nutrient digestibility, improving rumen fermentation patterns, and increasing rumen bacterial community diversity in multiparous Suffolk sheep.

## 1 Introduction

Suffolk sheep are commonly selected as sire breeds for producing hybrid mutton sheep owing to their superior meat yield and quality (Chen et al., 2022). Hu sheep, renowned for their year-round estrus, are commonly utilized as the dam breed in hybrid mutton sheep breeding (Yao et al., 2021). Crossbred multiparous Suffolk sheep, derived from the breeding of Suffolk and Hu sheep, exhibit high reproductive rates and meat production. Xinjiang, situated in China’s arid and semi-arid climatic zone (Geng, 2022), is characterized by long, cold winters and dry summers. During season transitions, the forage supply is often insufficient. In recent years, the rapid development of intensive breeding farms in Xinjiang has exacerbated forage shortages, resulting in livestock experiencing mild hunger during certain periods.

Feed costs constitute a major restrictive element in the advancement of intensive animal breeding operations, as feed intake directly influences the overall dietary expenses. Adjusting feed intake is a widely used strategy to manage production costs (Piles, 2017). Previous research indicates that feed restriction at 70–90% is classified as moderate, whereas restriction at 60% or below is severe, resulting in cessation of animal growth (Gidenne et al., 2009; Lourenço et al., 2022). During production, mild feed restriction reduces feed costs and enhances feed conversion rates during ad libitum feeding periods (Gibbs et al., 2015; Birolo et al., 2016). Restrictive feeding decreased dairy cow feed intake by 12.8% and energy-corrected milk (ECM) yield, while enhancing feed efficiency (Ben Meir et al., 2019). For Santa Inês sheep, increasing the degree of feed restriction significantly reduces the average daily gain (ADG) but expands the absorption area of the rumen and intestines (Lima et al., 2022). Lambs (36 kg) subjected to 40% feed restriction for 5 weeks, followed by 4 weeks of ad libitum feeding, exhibited no significant effect on yield, while maintaining economic viability (Abouheif et al., 2013). Feed intake levels enhance the feed conversion rate (FCR) by affecting feed digestibility, retention time, and nutrient absorption. Therefore, quantitative feed restriction could serve as a viable approach to enhance the economic efficiency of large-scale farming by saving forage.

Probiotics are safe and efficient green feed additives that can improve growth performance, promote rumen fermentation (Leyva-Diaz et al., 2021), and serve as alternatives to antibiotics (Zhang et al., 2016). Common probiotics include *Lactobacillus*, *Bifidobacterium*, yeast, and their cultures. Among these, yeast is commonly utilized in animal husbandry because of its low economic production costs, abundant availability, and significant effects on ruminant growth performance (Zhang et al., 2022). Yeast culture (YC), produced through the fermentation of *Saccharomyces cerevisiae*, is rich in amino acids (AA), crude proteins, crude fat (EE), and polysaccharides. YC improves ruminant growth performance by enhancing Dry mattera (DM), organic matter (OM) and apparent crude protein (CP) digestibilities (Haddad and Goussous, 2005), increasing the rumen pH (Dias et al., 2018), promoting rumen epithelial development (Qi and Wang, 2024), and increasing the abundance of cellulolytic bacteria (Halfen et al., 2021) and *Lactobacillus* in the rumen microbiota (Carpinelli et al., 2021).

Although prior studies have investigated the effects of feed restriction on sheep feeding behavior, methane emissions, and wool quality (Behrendt et al., 2021; Muir et al., 2021; De Brito et al., 2024), research on YC supplementation effects on rumen fermentation and microbial community structure under restricted feeding conditions is limited. Recent research shows that 0.5–2% YC supplementation under heat stress or high-concentrate diets increases rumen pH and concentrations of propionate and butyrate, enhances microbial diversity (Chao1 and Shannon indices), and increases the abundance of functional bacteria (e.g., *Prevotella*, *Butyrivibrio*, *Megasphaera*), thus improving production performance (Chen et al., 2024; Qi and Wang, 2024; Wang et al., 2024; Zhang et al., 2024). However, the effects of YC supplementation under feed restriction in sheep remain unexplored. Other research indicates that supplementing 20–40 g/d YC promotes rumen development and enhances the abundance of *Ruminococcaceae*, *Lachnospiraceae*, and *Ruminococcus* (Wang et al., 2023). Under feed restriction, YC improves production performance in sheep by optimizing rumen fermentation and microbial composition. This study is the first to assess whether YC supplementation mitigates body weight loss in multiparous Suffolk sheep under feed restriction, while systematically evaluating its effects on rumen fermentation [Volatile fatty acids (VFAs) and pH], microbial community interactions, feed efficiency, and production performance. This study aims to comprehensively evaluate YC’s potential to enhance production efficiency and nutrient utilization in sheep under feed restriction, providing a theoretical foundation for its application in precision feeding systems.

## 2 Materials and methods

### 2.1 Ethics statement

The planning and execution of the experimental protocol and animal care procedures obtained approval from the Animal Ethics Committee of Shihezi University (Shihezi, China; Approval No. A2023-241), with all operational parameters strictly aligned to standardized guidelines outlined in the National Research Council’s *Guide for the Care and Use of Laboratory Animals* (8^th^ revised edition).

### 2.2 Animals, diets, and experimental design

The experiment was conducted at the Longrui Probiotic Forage Feed Plantation Co-operatives, located in the Fifth Group of Xiaotuguli Village, Hutubi County, Changji City. For the formal experiment, 30 purebred multiparous Suffolk rams, consistent in generation, in good body condition, aged 4 months, and weighing 22 ± 0.5 kg, were selected from the 181st Corps of Altay Prefecture, Xinjiang Province. After passing quarantine, the rams were transported to the experimental site and subjected to acclimatization. Standardized prophylactic immunization protocols against foot-and-mouth disease (FMD) and peste des petits ruminants (PPR) were implemented following confirmation of stabilized nutritional intake parameters.

A single-factor experiment was conducted, with the experimental sheep randomly assigned to three groups: the ad libitum feeding group (AL), the intake restriction group (IR), and the intake restriction with 30 g/d YC group (IRY). Each group had 10 replicates. The experiment lasted for 67 days, including 60 days of formal testing and 7 days of preliminary testing. The apparent digestibility of nutrients trial began on day 52 of the formal experiment, lasting 7 days, with a preceding 3-day acclimation phase.

Feeding protocols involved twice-daily administration at standardized intervals (0900 and 1800 h), with ad libitum water accessibility. YC was supplemented at 30 g/d per sheep, based on the recommended dosage (0.5–1.5 g/kg initial body weight) from Weifang Shengyi Biological Feed Co., Ltd. and thoroughly mixed with TMR before each feeding. Daily records of feed offered and refused were kept for the AL group. The daily feeding amounts for the IR and IRY groups were adjusted based on the average daily intake of the AL group over the first 3 days. Each experimental sheep was individually accommodated in a pen with dimensions of 1.5 × 1.7 × 1.7 cubic meters. The pens were cleaned daily, and thorough disinfection was carried out every 10 days inside and outside the pens. Based on the National Research Council (2007) standard for 22 kg body weight and a daily weight gain of 300 g/d, a total mixed ration (TMR) was formulated with a constant concentrate-to-forage ratio of 60:40. The nutritional composition and formulation details of the experimental diet are presented in Table 1.

**Table 1:** The composition and nutrient levels of basal diets (air-dry basis).

Ingredients	Contents (%)	Nutrient components	Contents
Alfalfa	40.00	Dry matter	90.55
Corn	36.00	NDF	46.31
Cotton meal	5.00	ADF	38.84
Soybean meal	5.00	Crude protein	16.80
Bran	4.90	ME (MJ/kg)^2^	11.62
Safflower seed meal	3.00	Ether extract	4.12
Premix^1^	1.80	Calcium	0.60
Wheat flour	1.80	Phosphorus	0.30
Soybean oil	1.50		
Brewer’s yeast	1.00		
Total	100.00		

^1^Premix provided the following per kg diet: VA 50000 IU; VE 1,000 mg; Fe 8 g; Zn 1.6 g; Mn 800 mg; Gu 160 mg; Se 0.3 mg; I 1.0 mg; Co 0.5 mg.

^2^ME was calculated value, the others were measured values.

Based on tabular net energy (NE) values for individual feed ingredients (National Research Council, 2007), the composition and nutrient levels of basal diets (air-dry basis) were formulated.

### 2.3 YC

The YC was provided by Weifang Shengyi Biological Feed Co., Ltd. (Shandong, China). The fermentation strain used is *Saccharomyces cerevisiae*, with a content of ≥ 10^8^ CFU/g. The composition includes AA, CP ≥ 20%, EE, crude fiber (CF), mannan ≥ 5%, ash, and moisture content ≥ 3%.

### 2.4 Sample collection and processing

During the formal experiment, each multiparous Suffolk sheep was weighed individually once a week, with weighing occurring before morning feeding. Before daily feeding, the feeds offered to and refused were recorded. For the multiparous Suffolk rams, calculations were performed to determine the dry matter intake (DMI), ADG, and feed-to-gain ratio (F/G).

Fecal samples were gathered via the rectal end collection method, where all feces from each multiparous Suffolk sheep over a period of 7 days were mixed and stored at −20°C. All dietary and fecal matrices underwent desiccation (65°C, 48 h) followed by 24 h rehydration. DM quantification involved gravimetric stabilization through iterative 65°C dehydration cycles. Processed samples were milled through 1 mm sieves, packaged in 150 × 220 mm hermetic containers, and maintained at 4°C. Each sheep has 10 mL of blood drawn from the jugular vein. The samples were centrifuged (3,000 rpm, 10 min, 4°C), with the resultant serum being partitioned into two 5 mL microcentrifuge tubes for preservation at −20°C.

For rumen fluid collection, five sheep were arbitrarily chosen from each group. Rumen fluid was collected through an oral stomach tube, with the first 100 mL discarded and the subsequent 100 mL collected. The fluid was filtered through four layers of gauze and aliquoted into two 50 mL sterile enzyme-free centrifuge tubes. One tube received a 25% metaphosphate solution, after which all samples were stored at −80°C.

### 2.5 Analysis of growth performance, apparent nutritional digestibility, and serum biochemical and immune indices

Based on body weight and the amounts of feed offered and refused, DMI, ADG, and F/G were calculated. Quantitative determinations of nutritional constituents (DM, OM, CP, neutral detergent fiber (NDF), acid detergent fiber (ADF)) in feed matrices were conducted following established analytical protocols. DM content was analyzed following AOAC methods (AOAC, 1990). CP analysis employed Kjeldahl nitrogen quantification technology (Kjeltec 8,200, FOSS Analytical, Denmark) following acid digestion protocols. NDF and ADF contents were measured according to previous studies (Soest et al., 1991). Digestibility was evaluated using the acid-insoluble ash (AIA) method.

The apparent digestibility (%) was computed through the AIA tracer technique using the following stoichiometric relationship:

Apparent nutritional digestibility (%) = 100 − [(A1 × A2)/(A3 × A4)] (Araújo Costa et al., 2013).

Where: A1 = Fecal nutrient concentration (%); A2 = Dietary AIA content (%); A3 = Dietary nutrient concentration (%); A4 = Fecal AIA content (%).

Serum biochemical indices (total protein (TP), albumin (ALB), globulin (GLO), Blood urea nitrogen (BUN), glucose (GLU), triglycerides (TG), total cholesterol (TC), alanine aminotransferase (ALT), aspartate aminotransferase (AST), alkaline phosphatase (ALP), high-density lipoprotein (HDL), and low-density lipoprotein (LDL) utilizing an automated clinical chemistry analyzer (ZY KHB-1280, Shanghai Kehua Bio-Engineering, China). Immunological parameter analysis involved enzyme-linked immunosorbent assay (ELISA) determinations for immunoglobulin (immunoglobulin A (IgA), immunoglobulin M (IgM), immunoglobulin G (IgG)) and proinflammatory cytokines interleukin-1 (IL-1), interleukin-6 (IL-6)) employing commercial assay kits (Nanjing Jiancheng Bioengineering, China) following manufacturer specifications.

### 2.6 Measurement of rumen pH, NH_3_-N, and VFAs

The pH of the collected rumen fluid was promptly assessed using a handheld pH meter (WTW pH 3,110, Xylem Inc. Munich, Germany) (Niu et al., 2025). The NH_3_-N concentration in rumen fluids was measured using an Amphenol-hypochlorite assay (Carpinelli et al., 2021). VFAs were determined using gas chromatography. The collected samples underwent centrifugation at 12,000 rpm for 10 min. Subsequently, the resulting supernatant was passed through a 0.22 μm water-phase filter membrane. Following filtration, the supernatant was subjected to extraction with an equivalent volume of ether. Finally, the extracted material was analyzed using a gas chromatograph (7890A, Agilent, United Kingdom). A 1 μL sample was injected into a gas-phase capillary column (30 m × 0.25 mm × 0.25 μm). The temperature program was set as follows: initial temperature at 60°C, increasing at 20°C/min to 220°C. The injector temperature was set at 250°C, and the detector temperature at 280°C. The sample was injected in splitless mode with a 50:1 split ratio. Helium was used as the carrier gas.

### 2.7 Analysis of rumen bacterial communities

Quantitative Real-Time PCR Analysis: Cryopreserved ruminal fluid specimens underwent microbial genomic DNA isolation employing commercial extraction kits under standardized protocols. Nucleic acid integrity was verified through spectrophotometric quantification (NanoDrop 2000, Thermo Fisher Scientific, United States). DNA quality was assessed by 1% agarose gel electrophoresis (Zhou et al., 2020). The universal primers 338F (5’-ACTCCTACGGGAGGCAGCAG-3′) and 806R (5’-GGACTACHVGGGTWTCTAAT-3′) were used in a specific PCR method to amplify the V3–V4 variable region of the bacterial 16S rRNA gene.

16S rRNA sequencing and analysis: Purified amplicons were normalized for equimolar library construction and sequenced (PE250) on an Illumina NovaSeq platform following manufacturer-specified workflows. Low-quality sequences with insufficient length or average score, as well as duplicate sequences and those containing ambiguous bases, were filtered and preprocessed. Raw sequencing data preprocessing involved adapter trimming using Trimmomatic (v 0.39), read merging via FLASH (v 1.2.11), and chimera removal with the UCHIME algorithm. High-fidelity sequences were clustered into operational taxonomic units (OTUs) at a 97% similarity threshold using QIIME2 (v 2021.11). A representative sequence was selected from each OTU using default parameters. OTU taxonomic classification was conducted by BLAST searching the representative sequences set against the Greengenes Database. Non-compliant OTUs were removed. OTU subsets were averaged and compared against the SILVA database (v138) using the BLASTn algorithm with a ≥70% bootstrap confidence threshold.

Sequence data were analyzed primarily using QIIME and R packages (v3.2.0). Alpha diversity indices at the OTU level, including Chao1, Observed Species, Shannon, and Simpson indices, were used to assess microbial community richness. Beta diversity was visualized using principal coordinate analysis (PCoA) based on Bray-Curtis distances. Taxa abundances at the phylum and genus levels were statistically compared among groups using Metastats. Linear discriminant analysis effect size (LEfSe) was used to identify differentially abundant taxa across groups with default parameters.

### 2.8 Raw data accession numbers

The raw data have been deposited in the NCBI Sequence Read Archive (SRA) under the accession number PRJNA1242813.

### 2.9 Statistical analysis

Initial statistical processing was carried out using Excel 2022, after which the normality of distribution for growth performance metrics, apparent nutrient digestibility values, serum biochemical and immune indicators, pH, VFAs, and NH_3_-N was evaluated via the Shapiro–Wilk test. Parametric data are presented as arithmetic means ± standard error of the mean (SEM). Statistical analysis was performed through one-way analysis of variance (ANOVA) using SPSS 22.0 software, with *post hoc* multiple comparisons conducted employing the Duncan test. A probability value of less than 0.05 (*p* < 0.05) was deemed to indicate statistical significance. Non-parametric statistical tests were applied to assess differences in rumen bacterial communities among three groups. Correlation coefficients were computed based on Spearman correlation distances, and visualization of these correlations was achieved through a heatmap constructed using Origin Pro 2021.

## 3 Results

### 3.1 Growth performance and economic benefits

There were no significant differences in the initial body weight among the three groups (*p* > 0.05). In the AL group, final body weight (FBW), net weight gain (NWG), DMI, and ADG were significantly higher than those in the IR and IRY groups (*p* < 0.05). Additionally, FBW, NWG and ADG were significantly higher in the IRY group compared with those in the IR group (*p* < 0.05). The F/G was significantly higher in the AL and IR groups compared with that in the IRY group (*p <* 0.05) (Table 2).

**Table 2:** The effect of adding YC on the growth and economic benefits of multiparous Suffolk sheep.

Items	Groups^1^			SEM^2^	*P*-value^3^
	AL	IR	IRY		
Initial body weight, IBW (kg)	22.15	22.21	22.24	0.045	0.930
Final body weight, FBW (kg)	38.74^a^	35.41^c^	36.65^b^	0.354	<0.001
Net weight gain, NWG (kg)	16.59^a^	13.20^c^	14.41^b^	0.415	<0.001
Dry Matter Intake, DMI (g/day)	1350.26^a^	1080.21^b^	1080.21^b^	14.877	<0.001
Average daily gain, ADG (g/day)	276.50^a^	220.00^c^	240.17^b^	6.968	<0.001
Feed: Gain, (F/G)	4.88^a^	4.91^a^	4.50^b^	0.111	0.001
Unit price of feed, CNY/kg	3.00	3.00	3.00	–	–
^4^Feed cost, CNY	291.66^a^	233.33^b^	233.33^b^	3.213	<0.001
Unit price of YC, CNY/kg	10.00	10.00	10.00	–	–
^5^YC cost, CNY	–	–	18.00	–	–
Purchase price, CNY/kg	25.00	25.00	25.00	–	–
^6^Gross profit, CNY	123.09^a^	96.67^c^	108.92^b^	4.491	<0.001

^1^AL: Ad libitum feeding group: IR: 20% restricted feeding group: IRY: 20% restricted feeding group with the addition of 30 g/d YC.

^2^SEM represents the standard error of the mean.

^3a,b,c^Mean values within a row bearing different superscript letters differed significantly (*p* < 0.05).

^4^Feed cost: Total feed consumption cost per sheep during the 60-day experimental period.

^5^YC cost: Total YC supplementation cost per sheep.

^6^Gross profit: 60-day profit per sheep, calculated as: Purchase price × NWG − Feed cost − YC cost.

When considering only feed and YC costs, while excluding labor, management, health, and disease prevention costs, the net profit per sheep in the IRY group was 12.25 CNY higher than that in the IR group (Table 2).

### 3.2 Apparent nutrient digestibility

There were no significant differences in DM, OM, or CP among the three groups (*p >* 0.05). However, the digestibility of NDF and ADF was significantly lower in the AL group compared with that in the IR and IRY groups (*p <* 0.05). Additionally, NDF and ADF digestibility were significantly higher in the IRY group compared with those in the IR group (*p <* 0.05) (Table 3).

**Table 3:** Nutritional digestibility of multiparous Suffolk sheep with or without the addition of YC.

Items^1^	Groups^2^			SEM^3^	*P*-value^4^
	AL	IR	IRY		
DM (%)	63.36	64.74	65.05	4.937	0.082
OM (%)	75.97	76.93	76.45	3.864	0.215
CP (%)	62.42	63.09	63.43	6.965	0.167
NDF (%)	63.21^c^	65.44^b^	66.27^a^	0.048	0.021
ADF (%)	58.16^c^	59.28^b^	61.36^a^	0.157	0.013

^1^DM, dry matter; OM, organic matter; CP, crude protein; NDF, neutral detergent fiber; ADF, acid detergent fiber.

^2^AL: Ad libitum feeding group; IR: 20% restricted feeding group; IRY: 20% restricted feeding group with the addition of 30 g/d YC.

^3^SEM represents the standard error of the mean.

^4a,b,c^Mean values within a row bearing different superscript letters differed significantly (*p* < 0.05).

### 3.3 Serum biochemical and immune indicators

The TP, GLU, and IgA concentrations in the AL group were significantly higher than those in the IR and IRY groups (*p <* 0.05). TP, GLU, and IgA concentrations in the IRY group were significantly higher than those in the IR group (*p <* 0.05) (Table 4).

**Table 4:** Effects of adding YC on serum biochemical and immune indices in multiparous Suffolk sheep.

Items^1^	Groups^2^			SEM^3^	*P*-value^4^
	AL	IR	IRY		
TP (g/L)	62.46^a^	57.84^c^	58.16^b^	0.685	<0.001
ALB (g/L)	28.99	26.18	27.73	0.357	0.623
GLB (g/L)	32.32	32.31	32.34	0.137	0.916
UN (mmol/L)	5.65	5.63	5.64	0.015	0.946
GLU (mmol/L)	4.23^a^	3.52^c^	4.06^b^	0.139	0.017
TG (mmol/L)	0.42	0.40	0.39	0.054	0.893
TC (mmol/L)	1.09	1.11	1.13	0.185	0.863
ALT (U/L)	7.54	7.51	7.51	0.244	0.817
AST (U/L)	10.57	10.52	10.55	2.914	0.793
ALP (U/L)	38.71	38.65	38.74	0.091	0.673
HDL (mmol/L)	0.84	0.80	0.85	0.146	0.780
LDL (mmol/L)	0.58	0.59	0.52	0.215	0.739
IgA (g/L)	1.42^a^	0.87^c^	1.04^b^	0.667	0.034
IgG (g/L)	10.34	10.53	10.52	1.432	0.689
IgM (g/L)	0.78	0.82	0.94	0.457	0.343
IL-6 (pg/mL)	25.21	26.13	26.63	0.475	0.283
IL-1β (pg/mL)	115.43	117.52	118.32	1.431	0.115

^1^TP, total protein; ALB, albumin; GLB, globulin; BUN, Blood Urea Nitrogen; GLU, Glucose; TG, Triglycerides; TC, Total Cholesterol; ALT, Alanine aminotransferase; AST, Aspartate aminotransferase; ALP, Alkaline Phosphatase; HDL, High-Density Lipoprotein; LDL, low-density lipoprotein; IgA, Immunoglobulin A; IgG, Immunoglobulin G; IgM, Immunoglobulin M; IL-6, Interleukin-6; IL-1β, Interleukin-1β.

^2^AL: Ad libitum feeding group; IR: 20% restricted feeding group; IRY: 20% restricted feeding group with the addition of 30 g/d YC.

^3^SEM represents the standard error of the mean.

^4a,b,c^Mean values within a row bearing different superscript letters differed significantly (*p* < 0.05).

### 3.4 Rumen fermentation parameters

Compared with that in the AL group, the rumen pH in the IR and IRY groups was significantly decreased (*p* < 0.05), but that in the IRY group was significantly higher than that in the IR group (*p* < 0.05). The IRY group significantly increased propionate concentrations compared to the AL and IR groups (*p* < 0.05). Moreover, the A:P ratio was significantly reduced in IRY group compared with that in the AL and IR groups (*p* < 0.05) (Table 5).

**Table 5:** Effects of adding YC on pH and VFAs in multiparous Suffolk sheep.

Items^1^	Groups^2^			SEM^3^	*P*-value^4^
	AL	IR	IRY		
pH	6.47^a^	6.27^c^	6.33^b^	0.082	0.037
NH_3_-N (mg/dL)	10.37	11.28	10.64	0.256	0.269
Acetate (mmol/L)	33.26	32.24	32.14	0.062	0.930
Propionate (mmol/L)	16.64^b^	16.35^b^	18.43^a^	0.394	0.026
Butyrate (mmol/L)	9.20	9.12	8.94	0.067	0.192
Acetate: propionate ratio (A: P)	2.00^a^	1.97^a^	1.74^b^	0.146	0.034
Propionate: butyrate ratio (P: B)	1.81	1.79	2.06	0.212	0.068
Acetate: butyrate ratio (A: B)	3.62	3.54	3.60	0.195	0.187
TVFAs (mmol/L)	60.36	60.44	60.46	0.32	0.940

^1^NH_3_-N, ammonia nitrogen; A: P, acetate: propionate; P: B, propionate: butyrate; A: B, acetate: butyrate; TVFAs, total volatile fatty acids.

^2^AL: Ad libitum feeding group; IR: 20% restricted feeding group; IRY: 20% restricted feeding group with the addition of 30 g/d YC.

^3^SEM represents the standard error of the mean.

^4a,b,c^Mean values within a row bearing different superscript letters differed significantly (*P* < 0.05).

### 3.5 Analysis of the diversity of the rumen bacterial communities

**Figure 1:**
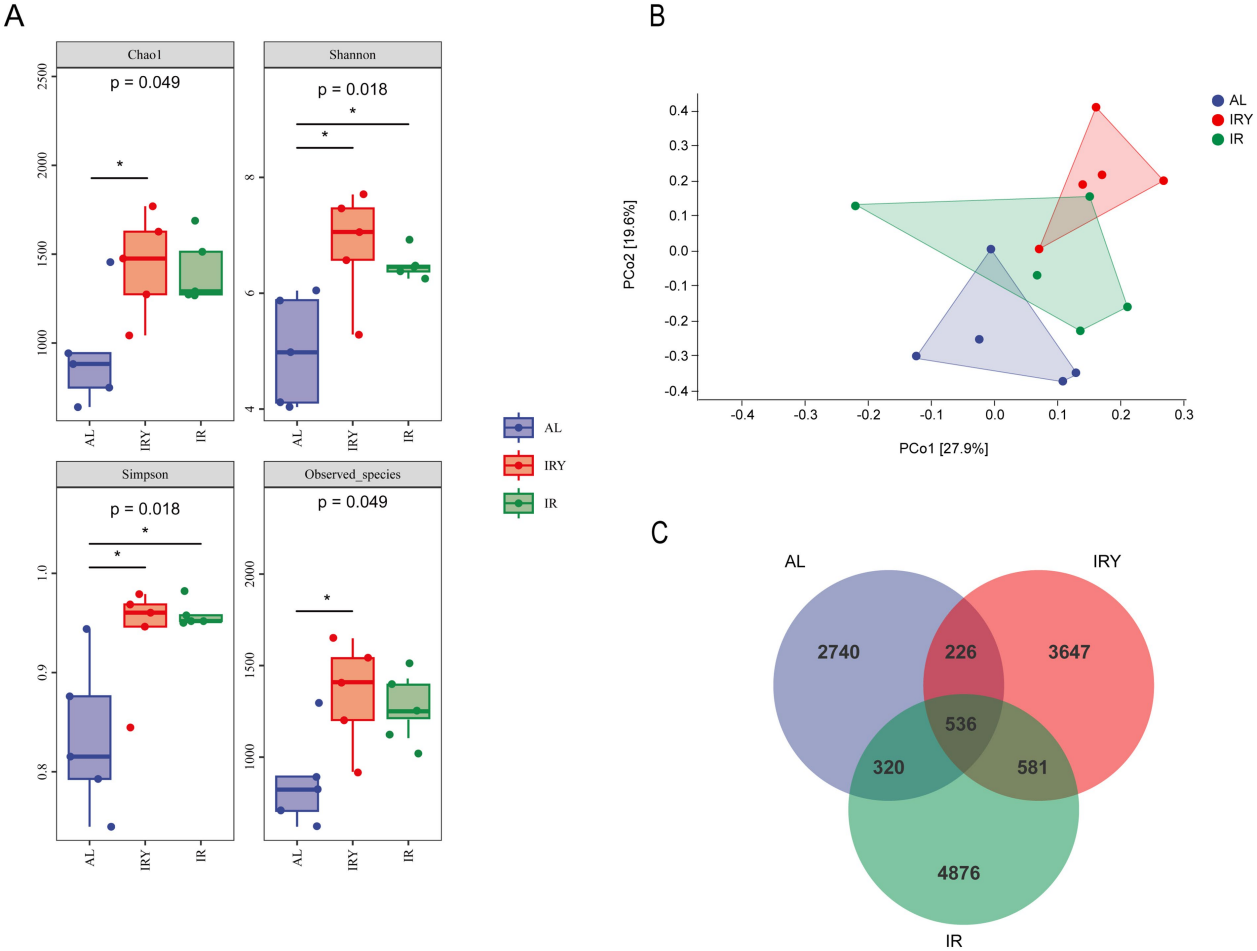
**(A)** Rumen alpha diversity indices (Chao 1, Shannon, Simpson, and Observed species) of the AL, IR, and IRY groups. **(B)** Beta diversity was assessed via principal coordinate analysis (PCoA) of the bacterial community structure in the AL, IR, and IRY groups. **(C)** Venn diagram.

A Venn diagram revealed 536 common amplicon sequence variants (ASVs) among all the groups. The AL, IR, and IRY groups had 2,740, 4,876, and 3,647 exclusive ASVs, respectively (Figure 1C). The analysis of rumen microbiota diversity and richness revealed that the Chao1 index of the IRY group was significantly higher than that of the AL group (*p* < 0.05). Both the Shannon and Simpson indices were significantly higher in the IRY and IR groups compared with those in the AL group (*p* < 0.05). In addition, the number of observed species in the IRY group was significantly higher than that in the AL group (*p* < 0.05) (Figure 1A). Principal coordinate analysis (PCoA) showed that the samples from the AL and IR groups were widely dispersed with little overlap, whereas samples from the IRY and IR groups were more concentrated with some overlap (Figure 1B). These results suggest that different feeding strategies affect the rumen bacterial composition in multiparous Suffolk sheep. Intake restriction modifies rumen bacterial composition, and the addition of YC further alters specific rumen bacterial communities.

### 3.6 Analysis of rumen bacterial phyla and genera

**Figure 2:**
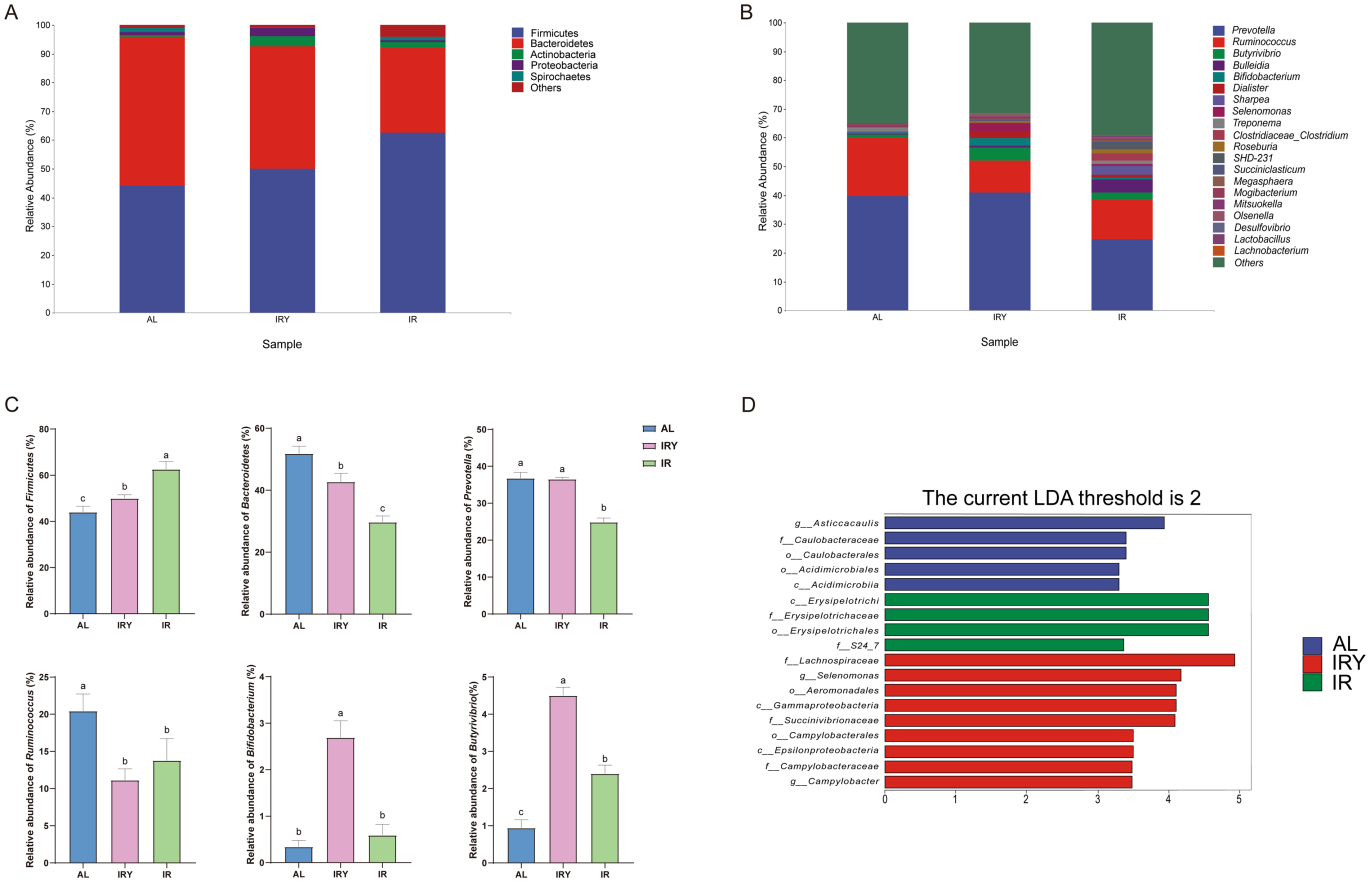
Rumen microflora composition of multiparous Suffolk sheep in the AL, IR, and IRY groups. **(A)** The top 5 phyla in the rumen. **(B)** The top 20 genera in the rumen. **(C)** Comparative differences of the relative abundance (%) of Firmicutes, Bacteroidetes, *Prevotella*, *Ruminococcus*, *Bifidobacterium*, and *Butyrivibrio* among the three groups. **(D)** LEfSe analysis of rumen bacterial flora in the AL, IR, and IRY groups. The biomarker taxa LDA score >2 of rumen microbiota in the AL, IR, and IRY groups. Higher LDA scores indicate a more significant role of bacteria in the phylogenetic microbial community. ^abc^Mean values bearing different superscript letters differed significantly (*P* < 0.05).

Firmicutes and Bacteroidetes were the dominant phyla in all three groups (Figure 2A). The relative abundance of Firmicutes was significantly higher in the IR and IRY groups compared with that in the AL group (*p* < 0.05). In contrast, the AL group had a significantly higher relative abundance of Bacteroidetes than the IRY and IR groups (*p* < 0.05). Additionally, the IRY group showed a significant increase in the relative abundance of Bacteroidetes compared to the IR group (*p* < 0.05; Figure 2C).

The dominant genera were *Prevotella*, *Ruminococcus*, *Butyrivibrio*, *Bulleidia*, and *Bifidobacterium* (Figure 2B). The average relative abundances of *Prevotella* in the AL, IRY, and IR groups were 39.67, 40.99, and 24.76%, respectively. There was no significant difference in the relative abundance of *Prevotella* between the AL and IRY groups (*p* > 0.05); however, both groups had significantly higher abundances of *Prevotella* compared with those in the IR group (*p* < 0.05; Figure 2C). The AL group exhibited the highest average relative abundance of *Ruminococcus* (20.43%), whereas the IRY and IR groups exhibited abundances of 11.13 and 13.77%, respectively. The AL group demonstrated a significantly higher relative abundance of *Ruminococcus* compared with that in the IRY and IR groups (*p* < 0.05), whereas no significant difference was observed between the IRY and IR groups (*p* > 0.05). Compared with that in the AL and IR groups, the IRY group exhibited higher relative abundances of *Butyrivibrio* and *Bifidobacterium* (*p* < 0.05; Figure 2C). Restricting the feeding of sheep not only increased the abundance of beneficial bacteria but also led to the production of probiotics such as *Selenomonas* (Figure 2B).

### 3.7 Differential species analysis

LEfSe analysis identified the key species of rumen microbiota among the three groups. When the threshold was set to 2 (Figure 2D), *Asticcacaulis* was identified as the featured bacteria in the AL group, whereas *Selenomonas* and *Lachnospiraceae* were the predominant bacteria in the IRY group.

### 3.8 Correlation analysis of the rumen bacterial communities and fermentation parameters

**Figure 3:**
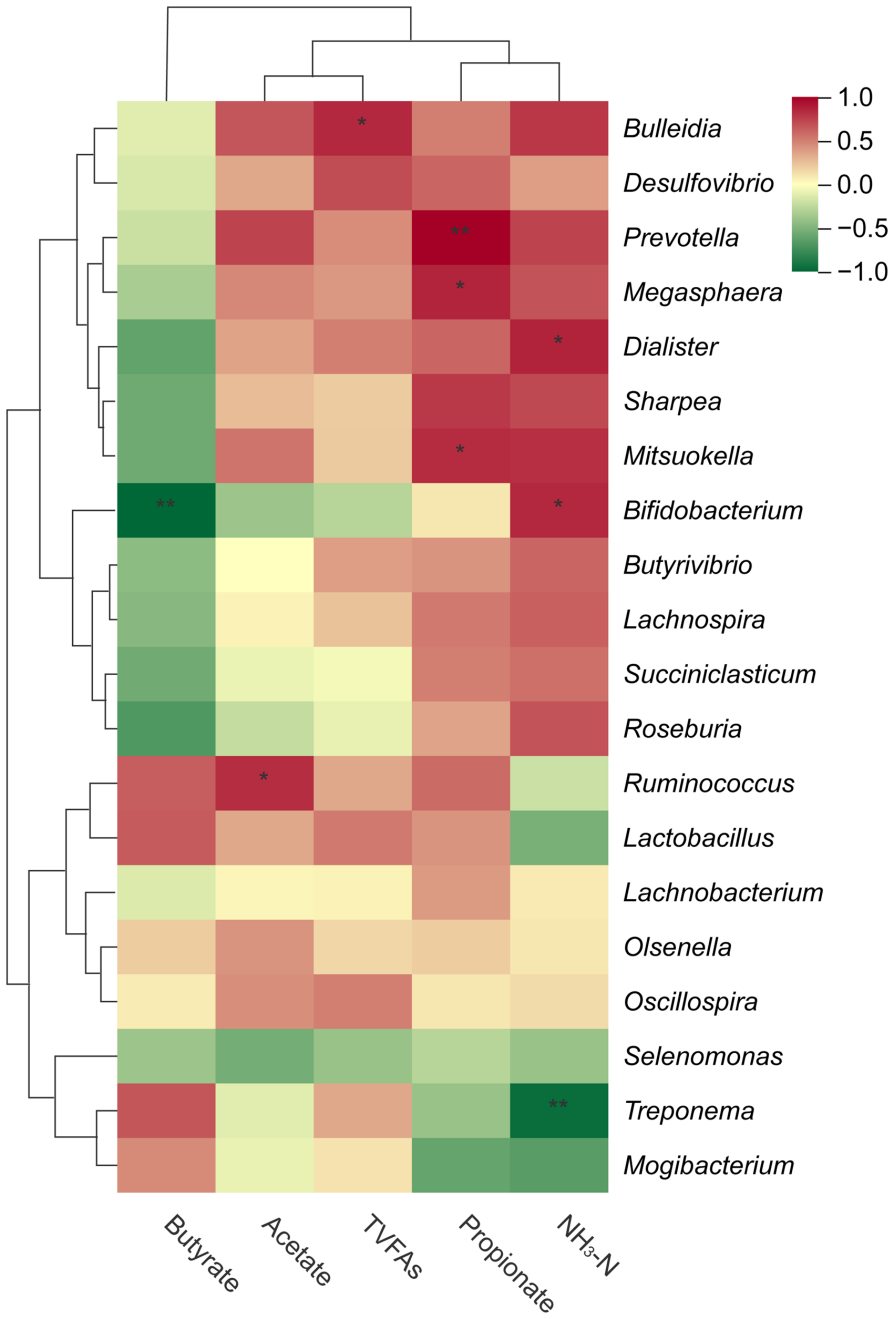
The heatmap visually represents the correlation between the top 20 bacterial genera in the rumen and VFAs, with red indicating a positive correlation, green indicating a negative correlation, and color intensity reflecting the correlation coefficient magnitude. *p < 0.05, **p < 0.01.

Spearman’s correlation analysis revealed that butyrate content was strongly negatively correlated with the abundance of *Bifidobacterium* (*p* < 0.01; Figure 3), NH_3_-N content was strongly negatively correlated with *Treponema* abundance (*p* < 0.001), while a positive correlation was observed between NH_3_-N content and *Bifidobacterium* (*p* < 0.05) and *Dialister* abundance (*p* < 0.05). Furthermore, propionate was strongly positively correlated with *Prevotella* (*p* < 0.01), and positively correlated with *Megasphaera* (*p* < 0.05), and *Mitsuokella* abundance (*p* < 0.05). Additionally, acetate levels were positively correlated with *Ruminococcus* abundance (*p* < 0.05), whereas *Bulleidia* abundance was positively correlated with TVFAs content (*p* < 0.05).

## 4 Discussion

### 4.1 YC improved the growth performance and economic benefits of sheep with restricted intake

Restrictive feeding is a widely applied strategy in poultry farming for controlling weight, enhancing egg production, and reducing feed costs (Metzler-Zebeli et al., 2019). This strategy is crucial in the sheep industry as it improves the FCR and serves as an effective transitional method during periods of feed scarcity in spring and winter, thereby providing economic benefits (Neto et al., 2011; Bezerra et al., 2013). However, the effects of different degrees of restrictive feeding on animal health vary significantly (Jiang et al., 2024). When the degree of restrictive feeding reaches 30%, the growth rate of sheep noticeably slows. At a restriction level of 60%, the weight of sheep almost stagnates (De Araújo et al., 2017). The energy required for animal growth is primarily derived from nutrient intake. When nutrient intake decreases, the energy produced by the animal is prioritized to maintain the basal metabolic rate (BMR), with any excess energy stored in fat or muscle. Restrictive feeding can lead to slower growth rates, and increasing levels of feeding restriction can result in growth arrest in animals (Almeida et al., 2006; Wang et al., 2021). As anticipated, the ADG of the IR group exhibited a significant reduction in this experimental study. The addition of YC to the IRY group’s diet increased ADG, this is consistent with the findings of previous studies (Wang et al., 2024) observation that YC supplementation enhances ADG in goats. Furthermore, this study found that the gross profit per sheep in the IRY group was 12.25 CNY higher than that in the IR group, demonstrating the economic feasibility of YC supplementation under restricted feeding conditions. Restrictive feeding induces mild hunger in animals, slowing rumen gastric emptying, and prolonging feed retention time, facilitating adequate digestion and absorption (Liu et al., 2021). This may explain the significantly higher digestibility of ADF and NDF in the IR group compared to the AL group. YC fermentation products, rich in AA, CP, and mannan, significantly enhance ADF and NDF digestibility, which are degraded into GLU. GLU, a primary energy source, is rapidly converted to ATP during energy deficiency to meet metabolic demands (Ortega Cerrilla and Mendoza Martínez, 2003), thus promoting animal growth performance (Yang et al., 2016). Thus, in this study, the improvement in ADF and NDF digestibility due to YC addition indicates their potential as contributing factors to the observed improved growth performance.

Ding et al. conducted a 30-day restrictive feeding trial on small-tailed Han sheep, followed by 60 days of ad libitum feeding, and they found that compensatory growth occurred during the ad libitum phase, which not only compensated for weight loss during the restrictive feeding period but also saved 18.4% of feed (Ding et al., 2016). Restrictive feeding does not necessarily harm animals, as animals can adapt their physiological and behavioral responses according to their nutritional status. Despite mildly restrictive feeding leading to a decrease in sheep weight (Table 2), it also resulted in the production of beneficial bacteria (Figure 2B), thereby facilitating compensatory growth.

### 4.2 YC improved serum biochemistry and immune indices

Serum TP levels reflect both the CP content in the diet and the efficiency of protein absorption in the intestines (Zhong et al., 2022). Within a certain range, higher TP values indicate better nutrient utilization by the animals (Ahmat et al., 2021). Restriction leads to reduced nutrient intake, thereby decreasing the availability of nutrients for digestion and absorption. In this experiment, the TP concentration in the serum of the IR group decreased, but the addition of YC significantly increased the TP concentration, indicating that YC effectively promoted protein digestion and absorption. The GLU concentration in the serum of ruminants was relatively low, which typically indicates insufficient energy availability or inadequate energy utilization by animals (Chai, 2015). Serum GLU concentrations in the IR group were significantly reduced. However, YC supplementation significantly increased GLU concentrations. NDF primarily comprises hemicellulose, cellulose, and lignin, whereas ADF consists of cellulose and lignin (De Souza et al., 2015). Cellulose and hemicellulose are degraded into GLU (Gírio et al., 2010), which may explain the increase in GLU concentration following YC supplementation and why the ADG in the IRY group exceeded that in the IR group. The addition of YC supplementation significantly increases villus length, width, and mucosal thickness in the rumen of calves, thereby improving their digestive and absorptive capacities (Qi and Wang, 2024). Changes in rumen morphology may contribute to increased TP and GLU concentrations, but this warrants further validation in future studies. Other studies have demonstrated that YC is associated with an elevated relative abundance of bacteria involved in protein and carbohydrate degradation, which enhances the digestion and absorption of proteins and GLU (Wang et al., 2023; Qi and Wang, 2024), leading to elevated serum TP and GLU concentrations. Within an appropriate range, GLU and TG can influence glucose-lipid metabolism in the body, and TG levels indicate the extent of fat absorption. In this study, restrictive feeding did not significantly alter the concentrations of TG or lipid metabolism indicators, such as TC and its predominant lipoprotein subclasses—high-density (HDL-C) and low-density (LDL-C) lipoprotein-cholesterol complexes, suggesting that restrictive feeding did not significantly affect lipid metabolism.

IgA, IgG, and IgM constitute the primary antibody classes within the immune system, each exhibiting distinct structural characteristics, sites of production, and functional roles in immune responses. The known reference range for serum IgA is 0.53–3.87 g/L (Costa et al., 2021). Moderate increases in IgA levels help enhance immune capacity. However, in the current investigation, it was observed that restrictive feeding led to a reduction in IgA concentration, indicating a decline in immune function in the experimental animals. In contrast, after the addition of YC, IgA concentration increased significantly, consistent with previous findings reporting elevated IgA levels in goats following YC supplementation (Qi and Wang, 2024). Mannan can activate immune cells (macrophages, dendritic cells (DCs), and complement components), thereby stimulating the immune system (Hagert et al., 2019). YC is rich in nutrients like mannan, which may activate the immune response and promote IgA production, potentially explaining the observed increase in IgA concentration. Additionally, other studies have indicated that the abundant AA, CP, and mannan in YC serve as essential substrates for the proliferation of immune cells and antibody synthesis (Demirgul et al., 2022), thereby enhancing immunity and contributing to an increase in IgA levels. Thus, they may be another key factor contributing to the increased IgA concentration observed following the addition of YC. No significant differences in IgG or IgM levels were observed among groups. Research shows that mannan oligosaccharide (MOS) specifically enhances local mucosal immune responses in the intestine (Yang et al., 2022). IgG is the primary antibody in secondary immune responses, IgM is mainly involved in primary immune responses to bacterial infections, and IgA is essential for mucosal immunity (Longbrake et al., 2021). This explains the lack of significant changes in IgG and IgM across groups.

### 4.3 YC altered the rumen fermentation patterns

Under normal physiological conditions, rumen pH in ruminants ranges from 6.0 to 7.0 (Grünberg and Constable, 2009), Maintaining rumen pH within the normal range is crucial for sustaining rumen fermentation. Both the feeding method, fiber and starch intake significantly affect rumen pH. A reduction in fiber intake leads to a decrease in the rumen pH (Zheng et al., 2020; Xiao et al., 2021; An et al., 2023), which may explain the observed decline in the rumen pH in this study. Increased starch intake, a rapidly fermentable carbohydrate, causes transient accumulation of VFAs, lowering rumen pH (Chen et al., 2016). Feed restriction increases the relative abundance of the lactate-utilizing bacterium *Megasphaera*. Although starch intake decreases, starch fermentation continues to produce lactate, which is rapidly converted to VFAs by lactate-utilizing bacteria (Yang et al., 2018), resulting in a decline in rumen pH. Ruminants secrete saliva during feeding, containing buffering substances such as bicarbonate and phosphate that neutralize acids produced during fermentation (Wang et al., 2016). Under feed restriction, reduced chewing leads to decreased saliva production, diminishing the availability of buffering substances and consequently lowering rumen pH. In this study, adding YC to the diet of restrictively fed sheep significantly increased the rumen pH, stabilizing it within the normal range and maintaining normal rumen fermentation function. In cattle, the addition of YC improves the rumen pH, stimulates rumen fermentation, and effectively mitigates the risks associated with dietary changes (Hansen, 2017). These findings align with the outcomes of the present investigation.

Acetate, propionate, and butyrate are the main VFAs (Bartel et al., 2022). The acetate-to-propionate ratio is an important indicator of rumen fermentation. Compared with the AL and IR groups, the addition of YC significantly decreased the A: P, shifting the fermentation pattern from the acetate to propionate type. Propionate serves as a precursor of GLU, and an increase in propionate levels can promote GLU synthesis. Therefore, propionate-type fermentation is favorable for the growth and fattening of livestock (Cui et al., 2021), which explains the improved growth performance of multiparous sheep following YC supplementation.

### 4.4 Effects of YC on rumen microbiota

#### 4.4.1 Ruminal bacterial diversity and phylum composition

Maintaining rumen microbial diversity is crucial for ruminant metabolism, immunity, and health. Comprehensive elucidation of the rumen microbiome composition and functional dynamics facilitates the formulation of targeted microbial management strategies to optimize nutritional conversion efficiency in ruminant production systems, improve animal health, and boost productivity (Yin et al., 2021). Rumen bacterial diversity is typically measured using alpha diversity indices, among which the Chao1 index reflects community richness, while the Shannon and Simpson indices reflect community diversity. In this study, the IRY group exhibited significantly higher community richness than the AL group, indicating that restrictive feeding combined with YC enhanced the rumen microbiome biodiversity. This observation aligns with prior research by Wang et al. (2021), demonstrating that elevated dietary energy intake in female Hu sheep correlated inversely with bacterial diversity indices.

Firmicutes and Bacteroidetes are the dominant phyla within the ruminal bacterial community of sheep (Zhang et al., 2021). In this experiment, neither restrictive feeding nor the addition of YC impacted the predominant phyla in the rumen of multiparous Suffolk sheep, but they significantly adjusted the ratio of Firmicutes and Bacteroidetes. Dietary YC supplementation resulted in an increase in Firmicutes abundance compared with that in the AL group. The primary genera within Firmicutes can decompose and utilize cellulose, thereby providing energy (Wang et al., 2019). The increased relative abundance of Firmicutes following YC supplementation contributed to elevated energy production in these animals. Conversely, excessive growth of certain genera within Bacteroidetes is closely associated with subacute rumen acidosis (SARA) (Tajima et al., 2001). In the present study, YC supplementation was associated with a diminution in the relative abundance of Bacteroidetes and a significant augmentation of the rumen pH, thereby enhancing rumen homeostasis. In summary, restrictive feeding and YC supplementation improved the rumen fermentation environment, provided more energy to the sheep.

#### 4.4.2 Composition of rumen bacterial genera and their relationships with rumen fermentation parameters

*Prevotella* is one of the most abundant genera in the rumen, and its relative abundance is affected by fiber intake (Wang et al., 2022). In this study, the reduced intake of ADF and NDF in the IR and IRY groups was associated with a reduced relative abundance of *Prevotella*. Additionally, *Prevotella* abundance showed a strong positive correlation with propionate concentration (Figure 3). Consequently, the decline in *Prevotella* abundance observed in the IR group corresponded to a decline in propionate levels (Jin et al., 2022; Pitta et al., 2022). However, following the addition of YC, the relative abundance of *Prevotella* increased significantly, accompanied by a marked increase in propionate concentration (Betancur-Murillo et al., 2022). *Butyrivibrio*, a cellulose-degrading bacterium, effectively degrades fibrous materials in feed. In this research, the significant increase in *Butyrivibrio* abundance after YC supplementation may have contributed to the improved digestibility of ADF and NDF in multiparous Suffolk sheep. Furthermore, *Bifidobacterium*, classified as *Lactobacillus*, can ferment and decompose carbohydrates to produce acetate and propionate, as well as provide energy for protein degradation, thereby increasing NH_3_-N production (Flint et al., 2008). In the present study, *Bifidobacterium* abundance was positively correlated with NH_3_-N concentration (Figure 3), and its relative abundance significantly increased after YC addition (Figure 2C), thereby promoting the growth performance of multiparous Suffolk sheep. LEfSe analysis revealed that the featured bacterium in the AL group was *Asticcacaulis*, but research on its role in the rumen is limited. In the IRY group, the featured bacteria included *Selenomonas* and *Lachnospiraceae*; *Selenomonas* is a lactate-utilizing bacterium capable of converting lactate to propionate, but the role of *Lachnospiraceae* in the rumen remains unclear. Overall, restrictive feeding and YC supplementation significantly altered the core bacterial community composition and abundance in multiparous Suffolk sheep.

Restrictive feeding has the dual benefits of reducing feed costs while promoting the growth of beneficial bacteria, such as *Selenomonas*, in the rumen bacterial community. *Selenomonas* can prevent rumen acidosis; when lactate accumulates and causes a decrease in rumen pH, *Selenomonas* migrates to the lactate accumulation area, exacerbating epithelial cell rupture and releasing intracellular materials, thereby mitigating the decline in rumen pH (Petri et al., 2020). *Selenomonas* abundance positively correlates with the live weight of beef cattle (Holman et al., 2024), and its increased relative abundance enhances the ADG of animals. In contrast, *Ruminococcus*, a cellulolytic bacterium, can ferment and decompose cellulose and resistant proteins, and its relative abundance was positively correlated with acetate concentration (Figure 3). However, there was no significant difference in the relative abundance of *Ruminococcus* between the IR and IRY groups. This indicates that YC, when used as a probiotic additive, has limitations in breaking down cellulose and carbohydrates.

## 5 Conclusion

This study explored the effects of YC as an additive on the growth performance of multiparous Suffolk sheep under intake restriction conditions. Under restricted feeding, YC supplementation significantly increased the abundance of *Butyrivibrio* and *Bifidobacterium* in the rumen. This enhanced rumen fermentation and nutrient digestibility, thereby improving the growth performance of multiparous Suffolk sheep. This study offers a novel strategy for addressing feed shortages and provides valuable theoretical support for ruminant nutrition and bacterial regulation.

## Data Availability

The data presented in the study are deposited in the NCBI Sequence Read Archive (SRA) repository, accession number PRJNA1242813.
